# Relationship of Circulating Irisin with Body Composition, Physical Activity, and Cardiovascular and Metabolic Disorders in the Pediatric Population

**DOI:** 10.3390/ijms19123727

**Published:** 2018-11-23

**Authors:** Leticia Elizondo-Montemayor, Gerardo Mendoza-Lara, Gustavo Gutierrez-DelBosque, Mariana Peschard-Franco, Bianca Nieblas, Gerardo Garcia-Rivas

**Affiliations:** 1 Tecnologico de Monterrey, Escuela de Medicina y Ciencias de la Salud, Ave. Morones Prieto 3000, Monterrey N.L. 64710, Mexico; germendoza14@gmail.com (G.M.-L.); gustavogtz92@gmail.com (G.G.-D.); marianapeschard@gmail.com (M.P.-F.); A00599747@itesm.mx (B.N.); 2 Tecnologico de Monterrey, Center for Research in Clinical Nutrition and Obesity, Ave. Morones Prieto 300, Monterrey N.L. 64710, Mexico; 3 Tecnologico de Monterrey, Cardiovascular and Metabolomics Research Group, Hospital Zambrano Hellion, San Pedro Garza Garcia P.C. 66278, Mexico

**Keywords:** irisin, pediatric, children, cardiovascular disease, nutrition, diet, body composition, metabolic syndrome, obesity, neonates

## Abstract

Exercise-induced irisin, a recently discovered myokine, has been linked to insulin resistance, obesity, and other diseases in adults; however, information in children is scarce and contradictory. We analyzed the limited evidence of irisin’s effects in children and adolescents, and its association with body composition, exercise training, cardiovascular risk factors, and metabolic diseases, as well as the results of dietetic interventions. Both positive and negative correlations between irisin concentrations and body mass index, fat mass, fat-free mass, and other anthropometric parameters were found. Likewise, contradictory evidence was shown associating irisin plasma levels with cardiovascular and metabolic parameters such as glucose, insulin resistance, and cholesterol and other lipid and fatty acid plasma levels in healthy children, as well as in those with obesity and the metabolic syndrome. Gender, puberty, and hormonal differences were also examined. Furthermore, important contradictory findings according to the type and duration of exercise and of dietetic interventions in healthy and unhealthy subjects were demonstrated. In addition, correlations between mother–infant relations and circulating irisin were also identified. This review discusses the potential role of irisin in health and disease in the pediatric population.

## 1. Introduction

Böstrom et al., 2012 [1] discovered irisin as a myokine derived from fibronectin type III domain-containing protein 5 (FNDC5) that is regulated by PGC1-α in mice. PGC1-α has several effects related to energy metabolism, including the activation of PPAR-γ that regulates the expression of the uncoupling protein 1 (UCP1) and thermogenesis in brown adipose tissue. It also stimulates the expression of messenger RNA (mRNA) of FNDC5, a muscle gene product that encodes a type 1 membrane protein. Irisin is a 112-amino acid polypeptide that is proteolytically cleaved from FNDC5, which undergoes glycosylation and is then secreted into the bloodstream. The original findings of Böstrom et al., 2012 [1] showed that exercise in humans induced circulating irisin, which activated browning and thermogenic genes in white adipose cells through UCP1, while it downregulated genes involved in white fat development. They hypothesized that irisin could play a role in increasing total body energy expenditure, reducing body weight, and improving obesity-related insulin resistance [1].

**Figure 1:**
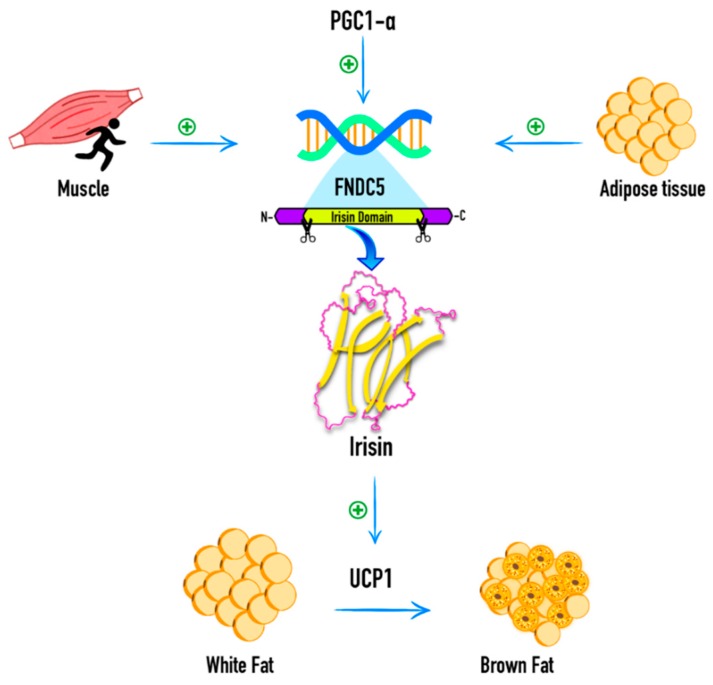
Irisin expression and function. Irisin is a myokine cleaved from fibronectin type III domain-containing protein 5 (FNDC5). Its expression is regulated by PGC1-α and it has been found to be secreted by adipose tissue and muscle. Irisin activates browning and thermogenic genes in white adipose cells through uncoupling protein 1 (UCP1), and downregulates genes involved in white fat development. PGC1-α: Peroxisome proliferator-activated receptor gamma coactivator 1-alpha. +: Positive influence.

More recent information indicates that irisin is also an adipokine with endocrine and autocrine functions secreted by white adipose tissue, and to a lesser extent, visceral adipose tissue (VAT) in the subcutaneous adipose tissue (SAT) [2]. Furthermore, irisin has also been found to be secreted by muscle. The expression of FNDC5 in muscle is related to SAT and VAT irisin levels and to the expression of FNDC5 and UCP1 genes in SAT. Muscle expression of the FNDC5 gene was 200-fold higher than that of adipocytes, indicating a relationship between muscle and adipose tissue functions in metabolic diseases. The correlation between irisin levels and FNDC5 expression in adipose tissue demonstrates a positive feedback mechanism for autocrine or paracrine production of irisin by adipose tissue, which further increases its capacity to metabolize glucose and fatty acids [3] (Figure 1).

Research has focused on finding mechanisms to explain the influence of irisin in the regulation of obesity, cardiovascular risk factors, metabolic syndrome (MS), and other related diseases. Irisin, named after the Greek messenger goddess Iris, has been linked to insulin resistance, obesity, exercise training, and cardiovascular and metabolic diseases in adults; however, the information on its role in children is scarce and contradictory. In this review, we discuss current knowledge in the pediatric population concerning irisin. The analysis also includes findings according to the type and duration of exercise in health and disease, those of dietetic and nutrition education interventions, as well as the influence of gender, puberty, and hormonal status on irisin plasma levels. In addition, the correlations between irisin and mother–newborn relationships are identified, along with the findings of irisin levels in plasma and tissues in other diseases in the pediatric population.

## 2. Association of Circulating Irisin with Body Mass Index and Body Composition

The associations of circulating irisin with body mass index and body composition parameters are shown in Table 1.

### 2.1. Body Mass Index And Anthropometric Parameters

The relationships between irisin, body mass index (BMI), and anthropometric parameters are still not completely understood. Literature findings have shown different correlations. Most clinical studies in adults have observed a positive correlation between irisin levels, weight, and BMI at both ends of the body weight spectrum [15,16,17]. Additionally, positive associations between irisin and body fat, waist circumference (WC), waist-to-hip ratio, and muscle mass have also been found [11,18,19].

However, the findings in children still differ substantially. In a study in Mexican children aged 2–6 years, plasma irisin levels were lower in the underweight group compared with the normal weight and obese groups. Irisin levels correlated positively with WC and BMI percentile, but after multiple linear regression analyses, the correlation remained only for the latter. The lower levels in the underweight children might be explained by the fact that this group presented the lowest total fat mass and body fat percentage, as well as the highest proportion of body muscle mass to fat mass [4].

Irisin levels have also been found to be higher in obese children compared with healthy children [5,20]. Other studies have shown a positive relationship of circulating irisin with BMI and waist-to-hip ratio in Turkish children [7] and with BMI and WC in Korean adolescents [8]. Elevated irisin was independently associated with the risk of obesity even after adjusting for age, sex, physical activity, puberty status, tryglicerides, low density lipoprotein-c (LDL-c), and HOMA-IR [8]. In addition, both before and after a physical activity intervention program, irisin levels have been shown to have a positive association with BMI [6].

In contrast, in prepubescent Korean children, those with obesity tended to exhibit a lower irisin concentration compared with normal-weight children [9]. There was also a significant inverse correlation between irisin and both BMI and WC; although after adjusting for age and gender, this relationship remained significant only for BMI in the normal-weight group, but not in the overweight/obese group. However, in a study that included obese and normal-weight children, irisin was significantly correlated with BMI at baseline, but after a lifestyle intervention program, changes in BMI were not related to changes in irisin levels [10], concluding that during childhood irisin levels are not related to weight status.

### 2.2. Muscle Mass and Fat Free Mass

The relationship between irisin levels and both muscle mass and fat free mass (FFM) is still contradictory. One study showed no correlation between irisin levels and FFM [7], while in others, a negative association was found between irisin and FFM in German children and adolescents [11], as well as in amenorrheic athletes (AAs), though not in eumenorrheic athletes (EAs) [12]. In contrast, other researchers have demonstrated a positive correlation between irisin and FFM [8]. Furthermore, in another study, the positive correlation between circulating irisin and FFM was lost after multiple regression analyses, while there was no relationship with muscle mass [4].

### 2.3. Fat Mass

Studies in Korean [8] and Turkish [7] children found a positive correlation between irisin concentration and both percent body fat and total fat mass. Jang et al., 2017 [8] considered body fat mass to be the most important independent factor in this relationship. However, no correlation was found between irisin plasma levels and total fat mass in Mexican children [4], or in athletic and non-athletic lean female adolescents [12].

These contradictory findings in children and adolescents have been attributed to the different body composition of children compared with adults and to the variations that occur during growth development and puberty resulting from the interplay between total fat mass, muscle mass, and the fat/muscle mass ratio. The lower irisin levels shown in underweight children might reflect an adaptive response to conserve energy, while irisin could play an ambiguous role in people who are obese. The higher levels in obese children suggest a compensatory role to increase subcutaneous brown adipose tissue and energy expenditure, and to improve obesity-related insulin resistance. Additionally, the higher irisin levels observed in obese people might be attributed to irisin resistance.

### 2.4. Bone Mineral Density

Irisin also appears to play a role in metabolic bone health. Several biomarkers secreted by adipose tissue, skeletal muscle, or bone may affect bone metabolism and bone mineral density (BMD). In adults, negative associations between irisin levels and BMD have been found in patients with previous osteoporotic fractures [21] and with an increased risk of hip fractures [22], probably due to the positive influence of irisin in bone quality, but not in bone mass [23].

In contrast, in a study of prepubescent Finnish children, irisin was positively and independently associated with BMD after adjusting for age and sex, and after controlling for lean and fat mass [13]. Similarly, a positive association between irisin levels and spine, femoral neck, and whole body bone density *Z*-scores, total and trabecular volumetric-BMD, and strength estimates has also been demonstrated in adolescent female athletes [12]. Thus, it is possible for irisin to induce the metabolic effects of brown adipose tissue on bone strength [24] and cortical thickness [25], as previously reported.

## 3. Association of Circulating Irisin with Physical Activity, Exercise Training and Dietetic Interventions

Table 2 shows the associations of circulating irisin with physical activity, exercise training, and dietetic interventions.

Studies in children concerning the changes in circulating irisin levels induced by physical activity have demonstrated inconsistent results and certain limitations. Regular, moderate physical activity appeared to maintain higher irisin levels in normal-weight adolescents compared with their sedentary counterparts; although this difference was not observed in overweight/obese adolescents [8]. However, another study showed a negative correlation between aerobic exercise and irisin levels in Mexican children alongside the entire BMI spectrum [4].

Varying results have also been demonstrated in dietetic, nutrition education, and physical activity intervention studies. Evidence suggests that acute intervals of aerobic physical activity seem to increase irisin concentrations in subjects. This was confirmed after observing a 2.23-fold increase in irisin levels after as little as 15 min of intense ergometer activity in normal-weight and obese German children [11]. A similar result was observed in the EXIT intervention trial in obese Canadian adolescents [26]. Circulating irisin was observed to increase by 60% after one 45-min session of aerobic exercise, but no change was observed after one 45-min session of weight-training exercise.

Long-term exercise and diet counseling seem to induce contradictory results. Irisin concentrations were found to be higher after one year of diet counseling and a combined endurance and resistance physical activity intervention program in overweight and obese German children, which resulted in decreased BMI [27]. No correlation between changes in BMI and irisin concentration was found, concluding that irisin levels were the direct result of exercise, and they were not influenced by changes in BMI. Similarly, in another one-year nutrition education and physical activity intervention consisting of a weekly low-intensity program, irisin levels were higher in obese children without significant weight reduction, but no changes in irisin levels were observed in obese patients with significant weight loss [10]. The authors suggested that unidentified confounders could explain these findings.

In contrast, after a 4 to 6-week nutritional education, psychological counseling, and daily exercise intervention that resulted in significantly lower BMIs, although inconsistent changes in irisin concentration were observed, most of the patients displayed decreased irisin levels [11]. The same authors evaluated a 3-year longitudinal comparison between children who performed regular, increased, or competitive levels of physical activity and found no difference in irisin levels among the groups. Similarly, a non-significant tendency towards decreased irisin levels was observed in healthy children after an 8-month physical activity program consisting of five weekly sessions of 25 min of moderate intensity exercise compared with overweight/obese children [6]. 

Finally, AAs aged 14–21 years showed lower irisin levels than EAs and non-athletes (NAs), even after controlling for age, body fat, and lean mass. Irisin concentration correlated with higher resting energy expenditure in all subjects. No difference was found between EAs and NAs, suggesting that irisin response is an adaptive reaction to preserve energy by decreasing resting energy expenditure and brown adipogenesis in AAs [12].

Interpretation of the results of studies concerning the association between irisin and physical activity is difficult because of the different physical activity regimens in regard to intensity, duration, regularity, number of sessions, the tools used to measure such activity, the combined dietetic interventions, and the studied pediatric populations. Irisin concentrations appear to increase after acute bouts of aerobic exercise, but not after long-term programs, especially in obese populations. Thus, the original hypothesis by Böstrom et al, 2012 [1] that irisin increases energy expenditure and decreases weight status in adults could apply to short bouts of exercise in normal-weight children and adolescents. The possibility that during long-term exercise programs, the body adapts to irisin thermogenesis has to be considered. As irisin seems to not increase in obese children even after weight loss, the loss of muscle mass that occurs during weight reduction might be responsible for the lack of change in irisin concentrations.

## 4. Association of Circulating Irisin with Cardiovascular and Metabolic Alterations

The association of irisin plasma levels with metabolic changes in insulin resistance, glucose, triglycerides, cholesterol, fatty acid composition, and other variables is shown in Table 3.

### 4.1. Insulin Resistance and Glucose Regulation

The relationship between circulating irisin and insulin resistance and impaired glucose metabolism in children is not completely understood. In a cross-sectional study in healthy children from Saudi Arabia, a negative correlation between plasma glucose and irisin was observed, but after adjusting for gender, this correlation remained significant in girls only [28]. In contrast, in obese pediatric populations, several studies have demonstrated a positive correlation of irisin levels with insulin resistance and glucose levels. In two Turkish cross-sectional studies, circulating irisin exhibited a positive correlation with glucose, insulin levels, and HOMA-IR in obese children compared with normal-weight ones [5,7].

However, in a cohort of overweight/obese and normal-weight Brazilian children, irisin levels showed a positive correlation with glucose and insulin levels and with HOMA-IR in both groups, though this correlation remained significant only for insulin after multiple logistic regression analyses [29]. Likewise, no significant differences in serum irisin levels between obese children with and without insulin resistance were demonstrated in Italian children [20]. Both studies attributed the lack of observed differences to the small number of subjects analyzed. Contradictory results were shown between two different longitudinal weight loss interventional studies in obese German children. In a study by Reinehr et al, 2015 [10], irisin levels were found to be higher in obese children with impaired glucose tolerance compared with those with normal glucose tolerance at baseline. Additionally, positive correlations were found between irisin concentration and insulin levels, HOMA-IR, and glucose tolerance tests. Although the positive correlations persisted after the intervention, irisin levels were not associated with changes in BMI; rather, a correlation was observed in children entering into puberty, probably due to the effects of insulin resistance related to hormonal changes. In contrast, a study by Bluher et al., 2014 [27] found a 12% increase in irisin levels after weight loss in overweight/obese subjects, but no correlation was found between irisin and insulin levels, HOMA-IR, BMI, or fasting glucose levels either at baseline or after the intervention.

Regarding the role of irisin in type 1 diabetes mellitus (T1DM) in children and adolescents, an Italian cross-sectional study found irisin levels to be higher in T1DM patients than in controls. Circulating irisin was even higher in patients with subcutaneous insulin infusion compared with the ones on multiple daily injection treatments. Furthermore, irisin showed a negative relation with HbA1c%, serum glucose, and years since the diagnosis of T1DM [14].

Because irisin levels were negatively correlated with glucose levels only in healthy girls, glucose could be an independent predictor of circulating irisin, attributing the difference between genders to dissimilarities in circulating hormone levels, or to the difference in brown adipose tissue quantity. Additionally, entry into puberty rather than BMI might be responsible for increased levels of irisin. Despite the contradictory correlations of irisin concentration with insulin resistance and glucose levels in the obese population, irisin has been proposed as a marker to differentiate obese children from normal-weight children. The increased irisin levels observed in obese subjects may represent the body’s compensation mechanism for the insulin resistance observed in this population by increasing insulin sensitivity. On the other hand, the increased irisin concentration may reflect a state of irisin resistance. The findings in T1DM children suggest that better metabolic control is related to higher irisin levels in this pediatric population.

### 4.2. Cardiovascular Risk Factors and the Metabolic Syndrome

Previous studies have reported a link between irisin and the MS in adults, but only a few studies have evaluated this relationship in the pediatric population. A significant positive correlation exists between irisin levels and BMI, WC, triglyceride levels, systolic blood pressure (SBP), and diastolic blood pressure (DBP); but an inverse correlation with HDL-c levels were observed in a study by De Meneck et al., 2018 [29]. However, after multiple regression analyses, the relationship remained significant only for WC. Similarly, a cross-sectional study by Jang et al., 2017 [8] found irisin levels to be positively correlated with SBP, WC, triglycerides, fasting plasma glucose, HOMA-IR, and LDL-c. Higher irisin levels increased the risk for obesity and MS by two-fold, even after adjusting for age, sex, physical activity, and puberty, but after adjusting for BMI, this odds ratio was lost. A positive correlation between circulating irisin levels and branched and aromatic amino acids was also found. The authors suggested that the metabolic actions of irisin start during childhood and that beta cell dysfunction and evolution towards metabolic diseases are driven by the interplay between circulating irisin and branched-chain amino acids, which highly influence adiposity, lipids, and glucose. 

In contrast, a cross-sectional study in prepubescent children found circulating irisin to be positively correlated with SBP and DBP only, but an inverse correlation with other components of the MS was demonstrated. After adjustments, irisin concentrations were found to be significantly lower in overweight and obese children with the MS compared with those without the MS. The authors proposed an irisin concentration of 15.43 ng/mL as a cutoff value for MS distinction, suggesting that irisin might be used as a biomarker for the MS [9]. 

The explanations for the contradictory role of irisin in metabolic diseases in children have yet to be clarified. Although myocytes are responsible for exercise-induced irisin secretion, in the context of increased adiposity, fat cells may be the primary source of high circulating irisin observed in some obese individuals with the MS. Increased fat mass may stimulate irisin production as a means to counteract new set points in energy balance. Conversely, a decrease in adipose tissue browning in obese individuals with the MS may be related to lower circulating irisin. Notably, in studies that showed positive associations between the MS and irisin, only anthropometric parameters such as BMI and WC, but not metabolic parameters, remained significant after adjustments, supporting the role of adipose tissue and its association with irisin in children. Furthermore, although it has been suggested that circulating irisin might regulate energy expenditure in adults with altered glucose metabolism, this compensatory mechanism could be limited in children, particularly before puberty, as a consequence of a proportionally lower muscle mass compared with adult populations. 

### 4.3. Adipocytokines

Contradictory results have also been demonstrated regarding the relationship between irisin levels and adipocytokines. Circulating irisin has been positively correlated with leptin, but negatively associated with adiponectin in both obese and normal-weight Korean children [8]. A negative correlation between irisin and adiponectin was also observed in obese and normal-weight Italian children [20]. In an interventional study, a positive correlation between irisin and leptin was observed before and after an 8-month physical activity program in normal weight and obese children. The decrease in leptin after the exercise intervention showed a strong association with the decrease in irisin levels across all BMI subgroups [6]. In contrast, two studies found no correlation between circulating irisin and leptin, adiponectin, or resistin levels, either at baseline or after lifestyle interventions [27,30]. Concerning inflammatory cytokines, positive correlations between irisin and TNFα and IL-6 were observed in a cross-sectional analysis of the PANIC study [27,30]. 

The negative association between irisin and adiponectin levels may indicate that in states of low energy expenditure, decreased adiponectin might stimulate a compensatory increase in irisin in order to increase energy expenditure. However, the positive association between irisin and leptin levels, which both decrease after a physical exercise intervention, supports the hypothesis that irisin is produced by adipose tissue. Since there are some inconsistent results, the interplay between irisin and adipocytokines needs to be further investigated. The association of irisin levels with TNFα and IL-6 suggests that irisin levels could be related to a proinflammatory profile. 

### 4.4. Fatty Acids Composition

Higher irisin levels have recently been associated with metabolically unfavorable fatty acid profiles in overweight and obese children, compared with normal weight ones, in a cross-sectional analysis from the ongoing PANIC study. In a subset of children, higher irisin levels were associated with polymorphism linked to an increased accumulation of hepatic triglycerides, suggesting that increased irisin levels may be intended to prevent lipid accumulation and progressive steatosis and fibrosis. Additionally, higher proportions of oleic acid, adrenic acid, and docosapentaenoic acid in plasma were associated with higher plasma irisin levels among overweight/obese children, which suggests that irisin is directly associated with increased activity of elongation and desaturation steps following the desaturation of linoleic acid. A possible association between plasma irisin and delta-6-desaturase activity in plasma cholesteryl esters was also found [31], which has previously been reported to be associated with insulin-resistant states [32]. Excess body fat could modulate these relationships through fatty acid-mediated cross-talk between metabolically active tissues.

### 4.5. Energy Intake and Expenditure

Recent studies have elucidated the interplay between new energy intake and expenditure regulators, such as oxytocin, which is involved in food intake regulation in the central nervous system. A positive correlation between circulating irisin and oxytocin levels was observed in AAs compared with EAs and NAs [33]. In contrast, in an obese population, irisin and oxytocin appeared to have opposite roles, as the first was found to be higher in obese and overweight pubescent children and adolescents compared with controls, while the opposite was found for oxytocin levels. Yet, a correlation between irisin and oxytocin was not studied by Binay et al., 2017 [7]. Thus, oxytocin signaling and the regulation of food intake may be more significant in high energy consumption situations, such as in AAs, while an inverse association is found in obesity.

## 5. Association of Circulating Irisin with Gender, Puberty, and Hormonal Status

Regarding the differences in irisin concentrations between genders, some studies in normal-weight subjects have concluded that circulating irisin levels were higher in lean girls than in lean boys. Noteworthy this difference between genders has not been observed in overweight or obese children [5,6,8,11,27,28]. As for puberty, a significant difference in irisin concentration between prepubescent and pubescent subjects was also observed in obese children. After one-year follow-up, an increase in irisin levels was found in five obese subjects that had begun puberty, compared with those who had not [10]. 

Exploring the relationship between irisin concentrations and hormonal levels, in a study of adolescent athletes, Singhal et al., 2014 [12] found no significant difference in sexual hormones between groups. However, irisin was found to correlate with a higher free androgen index in AAs, while a positive correlation between irisin and estradiol was observed in NAs. The meaning of these findings is not yet understood. 

Most studies have concluded that gender differences in irisin concentrations in children and adolescents are not due to puberty status, but rather to total adipose tissue, brown adipose tissue, and metabolic activity, with girls having a higher body fat mass than boys. However, a contradictory conclusion was obtained in one of the previous studies stating that in the obese population, entry into puberty was the main factor involved in the increased irisin levels. 

## 6. Association of Circulating Irisin with Mother–Offspring Relationship and Gestational Age in Neonates

Circulating levels of irisin in newborns are believed to be maternally inherited. In a cross-sectional study in Arab families, circulating irisin was found to be an inheritable trait between mother and offspring [34]. Others have found significantly lower irisin levels in newborns than in mothers. In a study of Mexican mother/newborn pairs with single, non-complicated pregnancies, only newborns from cesarean sections presented lower irisin concentrations than their mothers compared with labor-born neonates [35]. Arterial cord blood total antioxidant capacity, IL-1β, and IL1-RA levels positively predicted newborn irisin concentrations. Maternal IL-13 negatively predicted offspring irisin levels, while IL-1β positively predicted newborn irisin concentrations. 

In another cross-sectional study in 70 pairs of newly-delivered Greek neonates and their mothers, irisin levels were also lower in the neonates [36]. A possible explanation for the higher irisin levels in labor-born neonates compared with cesarean-born ones could be related to physical stress during labor possibly mimicking physical activity. Additionally, differences in neonate and adult muscle mass could explain the lower irisin concentrations found in neonates compared with their mothers. 

Associations between irisin concentrations and gestational age in newborns have been demonstrated. Comparing newborns from different gestational ages, many studies have found higher irisin levels in term infants compared with preterm newborns, as well as positive correlations with birth weight Z-scores. Measurement of irisin in umbilical cord blood has shown that irisin levels are lower in small for gestational age (SGA) newborns compared with appropriate for gestational age (AGA) and large for gestational age (LGA) neonates [36,37,38]. In contrast, in a study of Caucasian women, a higher irisin level in preterm infants compared with term infants was found [39]. Irisin levels were also significantly higher in maternal blood compared with umbilical cord blood. In intrauterine growth restriction (IUGR) subjects, fetal irisin concentrations were found to be significantly lower, compared with AGA controls and LGA neonates [40]. In the LGA group, fetal irisin concentrations were positively correlated with fetal insulin levels. 

The lower irisin concentration in SGA and IUGR newborns might be attributable to their smaller muscle mass. It has been previously shown that IUGR neonates have impaired skeletal muscle growth [41], while SGA neonates have a smaller skeletal muscle mass [42] and total body fat percentage [43] compared with AGA newborns. Since irisin is needed for non-shivering thermogenesis, which is crucial for the adaptation of the newborn to the postnatal environment IUGR, and SGA neonates with low irisin levels might be predisposed to hypothermia at birth [40]. In addition, IUGR, together with LGA neonates, are at high risk for obesity and metabolic disorders, as well as for alterations in fetal adipose tissue development and hormonal dysfunctions [44]. These facts might explain the positive correlation between fetal irisin and insulin levels observed in the LGA group. Therefore, irisin might be an important metabolic factor during very early stages of life that may render these neonates susceptible to insulin resistance and the MS later in life. 

## 7. Association of Circulating Irisin and Other Diseases

Although irisin has been studied in a few other organs and diseases in the pediatric population, its role remains unclear. High irisin immunoreactivity evaluated through immunohistochemistry was demonstrated in acute appendicitis biopsies from Turkish children. Positive correlations of irisin levels with urine, saliva, and serum, as well as with total white blood count, neutrophil percentage, and reactive C-protein have also been found. Irisin was higher in all samples from patients with appendicitis, compared with controls. In the post-operative period, irisin concentration reached baseline levels 72 h after surgery, suggesting that irisin secretion could be increased in response to the acute inflammation of appendix tissues [45].

Irisin was also studied in Egyptian children with epilepsy. Children with idiopathic epilepsy presented higher irisin levels compared with controls [46]. Plasma irisin showed a positive correlation with the severity of seizures and the duration of the disease, probably playing a role as a predictor of uncontrolled seizures. The authors hypothesized that the elevated irisin levels may play a protective role against the hypoxic effects of seizures, as has been demonstrated by Zhao et al., 2016 [47] and Mazur-Bialy et al., 2017 [48] in animal models.

Finally, in a study of prepubescent patients with Turner syndrome who underwent recombinant human growth hormone (rhGH) therapy, a significant increase in irisin levels was found after treatment, although no relationship between irisin and IGF-1 was observed before or after therapy [49]. Negative associations between irisin levels and metabolic parameters were found, while a positive association of irisin with HbA1c was identified. These results suggest that in treatment-naïve Turner syndrome patients, who are predisposed to the MS, the physiological role of irisin may be disrupted and that rhGH therapy may restore it.

**Figure 2:**
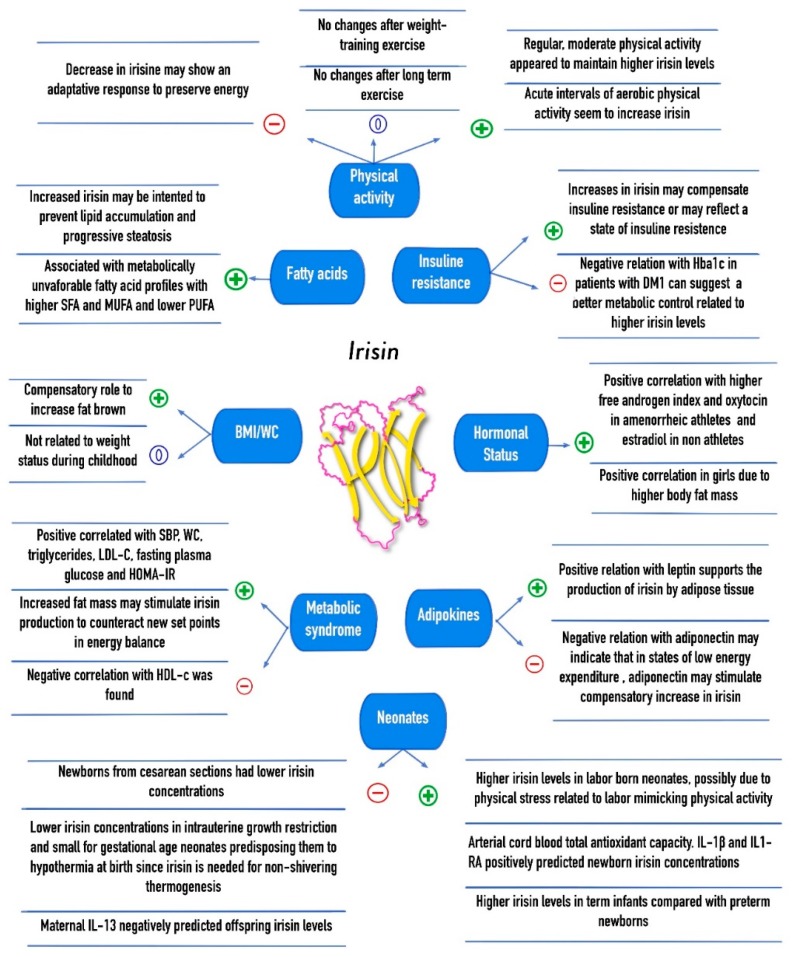
Association of irisin concentrations with cardiovascular, metabolic, and anthropometric parameters, physical activity, and mother-infant relations in children. +: Positive correlation; −: Negative correlation; 0: No correlation; SFA: Saturated fatty acids; MUFA: Monounsaturated fatty acids; PUFA: Polyunsaturated fatty acids; SBP: Systolic blood pressure; WC: Waist circumference; LDL-c: Low density lipoprotein cholesterol; HDL-c: High density lipoprotein cholesterol; HOMA-IR: Homeostatic model assessment of insulin resistance; DM1: Diabetes Mellitus type 1; IL13: Interleukin 13; IL-1β: Interleukin 1 beta; IL1-RA: Interleukin 1 receptor antagonist.

Figure 2 summarizes the associations of plasma irisin levels with body composition, cardiovascular risk factors, the metabolic syndrome, diet and physical activity interventions, as well as neonates and infant–mother correlations in the pediatric population.

## 8. Conclusions

The role of irisin as a regulator of body composition, and cardiovascular and metabolic diseases, as well as its correlation with physical activity and dietetic interventions has been understudied in the pediatric population, and it is still poorly understood. Contradictory findings have been found regarding the association of irisin with BMI, WC, fat mass, muscle mass, cardiovascular risk factors, insulin resistance, fasting glucose, and lipid levels, as well as its role in obesity and the MS. Irisin levels have been found to differ by gender in lean children, but not in obese ones. Controversial hypotheses to explain these findings have also been explored.

Irisin might represent an adaptive response to preserve energy in children with decreased muscle and fat mass, such as those who are underweight, and in SGA and IUGR neonates. Meanwhile, in children and adolescents with obesity, cardiovascular risk factors and the MS, irisin might either increase energy expenditure through thermogenesis, or it may represent an insulin-resistant state, especially considering the negative association of irisin with adiponectin and its positive association with leptin. Furthermore, irisin has even been associated with a proinflammatory profile.

Increased irisin levels during short bouts of aerobic exercise may only represent increased energy expenditure, but the lack of response during long term regimens, which even include nutrition and diet counseling, may be attributed to an adaptive thermogenesis. Further research in the pediatric population is required to confirm the association of irisin concentration with body composition parameters, physical activity, and cardiometabolic diseases, as well as to elucidate the role of irisin and its underlying mechanisms as a regulator of the metabolic state.

## Figures and Tables

**Table 1:** Association of circulating irisin with body mass index and body composition.

Author	Sample	BMI%/BMI Z-Score	Body Composition Measurement	FM	MM%/FFM	BFP/BFM	WC/WHR	Others
Elizondo-Montemayor 2017 [4]	*n* = 40 (20 boys) Mexico 6–12 y-o. UW (*n* = 5), NW (*n* = 5), OW (*n* = 5), OB (*n* = 5).	+	Fat mass = [(weight-kg) × (body fat%)]/100 Body muscle= (height-cm) [0.264 + (0.0029 × MUAMA-cm^2^)] FFM = (weight-kg − (weight-kg × body fat%)) PBF:Bioelectric impedance analysis (TANITA TBF 300)	0	MM (−)	0	WC (+)	N/A
Catli 2016 [5]	*n* = 66 Turkey 8-15 y-o. OB (*n* = 20 (20 boys)), NW (*n* = 30 (16 boys)).	0	Bioelectric impedance analysis (TanitaBC-41)	0	N/A	+	WC (0)	SBP (0), DBP (0)
Palacios-González 2015 [6]	*n* = 85 (40 boys) —Mexico 8–10 y-o. NW (*n* = 25), OW (*n* = 23), OB (*n* = 37).	+	N/A	N/A	N/A	N/A	N/A	N/A
Binay 2017 [7]	*n* = 120 Turkey 10–18 y-o. OB (*n* = 90), NW (*n* = 30).	+	Bioelectrical impedance analysis (BC-418MA Tanita Segmental Body Composition Analyzer)	+	0	+	WHR (+)	SBP (+)
Jang 2017 [8]	*n* = 618 (316 boys) Korea 12–15 y-o. NW (*n* = 370), OB (*n* = 248).	+	Bioelectrical impedance analysis (BC-418; Tanita)	+	+	+	WC (+)	N/A
Shim 2018 [9]	*n* = 96 (56 boys) Korea 6–10 y-o. NW (*n* = 54), OW (*n* = 16), OB (*n* = 26).	-	N/A	N/A	N/A	N/A	WC (-)	SBP (+), DBP (+)
Reinehr 2015 [10]	*n* = 60 Germany 10–15 y-o NW [*n* = 20 (10 boys)], OB (*n* = 40 (20 boys)).	0	N/A	N/A	N/A	N/A	N/A	DBP (+)
Löffler 2015 [11]	*n* = 105 (46 boys) Germany NW (*n* = 20), OW/OB (*n* = 64) 8–21 y-o and OB (*n* = 58 (23 boys)) 7–17 y-o.	BMI (+)	Bioimpedance analyses (Nutriguard-MS) Fat free mass and body cell mass (NutriPlus Software)	0	FFM (+) adults FFM (−) children	0	WHR (+)	SBP (0) DBP (0)
Singhal 2014 [12]	*n* = 85 women (81 Caucasian and Asian, 11 mixed- race, 5 Black) 14–21 y-o. AA (*n* = 38), EA (*n* = 24), NA (*n* = 23).	N/A	Dual energy x-ray absorptiometry (DXA)	0	(+) in all athletes (+) in AA (0) in EA	N/A	N/A	Spine BMD Z-score (+) Whole body BMD Z-score (+) Total vBMD (+) Trabecular vBMD (+)
Soininen 2018 [13]	*n* = 472 (245 boys) Finland 6–8 y-o.	N/A	MM, FM, PBF, BMD = Lunar Prodigy Advance DXA device	N/A	N/A	N/A	N/A	BMD (+) all children, not with boys and girls separately
Faienza 2018 [14]	*n* = 127 Italy 6–16 y-o. DM1 [*n* = 96 (41 boys)] 8–16 y-o, CO (*n* = 35 (21 boys)) 6–12 y-o.	(+) in patients with SCII	N/A	N/A	N/A	N/A	N/A	(+) with BTT-Z score, PTH, osteocalcin (−) with serum calcium, 25(OH) vitamin D, DKK-1, and sclerostin

y-o (years old), AA (amenorrheic athletes), BFM (body fat mass), BFP (body fat percentage), BMD (bone mineral density), DBP (Dystolic blood pressure), EA (eumenorrheic athletes), FFM (fat free mass), FM (fat mass), MM (muscle mass), MUAMA (mid-upper arm muscle area) PBF (percent body fat), NA (non-athletes), N/A (not available), NW (normal weight), OB (obese), OW (overweight), SBP (Systolic blood pressure), UW (underweight), vBMD (volumetric bone mineral density), WC (waist circumference), WHR (waist–hip ratio), + (positive correlation), − (negative correlation), 0 (no correlation).

**Table 2:** Association of circulating irisin with physical activity and exercise training and dietetic interventions.

Author	Sample	Intervention		Correlation	Results
Jang 2017 [8]	*n* = 618 (316 boys) Korea 12–15 y-o. NW (*n* = 370), OB (*n* = 248)	No Intervention		+	In NW girls
				+	In NW active
Elizondo-Montemayor 2017 [4]	*n* = 40 (20 boys) Mexico 6–12 y-o. UW (*n* = 5), NW (*n* = 5), OW (*n* = 5), OB (*n* = 5).	Reported aerobic exercise (days per week and hours per day)		−	With days per week
				-	With hours per day
Löffler 2015 [11]	*n* = 29 (11 boys) Germany 8–21 y-o. OB (*n* = 10 (2 boys)).	15-min maximum cycle ergometer		+	In all subjects
	*n* = 58 OB (23 boys) Germany 7–17 y-o.	4–6 weeks of nutritional and aerobic training		+/−	In boys
	*n* = 88 Germany 11–12 y-o. CO [*n* = 29 (12 boys)], IN (*n* = 34 (20 boys)), CS (*n* = 25 (16 boys))	Intervention group increased one unit of sports activities for 3 years		0	No difference among groups
Blizzard LeBlanc 2017 [26]	*n* = 11 OB (6 boys) Canada 15–16 y-o.	Acute bouts of exercise:	Aerobic: 45 min at 60% HRR	+	In all subjects
			Resistance: 45 min at 60–65% 1RM 12–15 reps	0	In all subjects
Palacios-González 2015 [6]	*n* = 85 (40 boys) Mexico 8–10 y-o. NW (*n* = 25), OW (*n* = 23), OB (*n* = 37).	5-min warm-up and 25-min aerobic activity at 75% HRMax, 8 months, 5 days/week		−	In all subjects
Blüher 2014 [27]	*n* = 65 Germany OW/OB (35 boys) 7–18 y-o.	39 session over 1 year of 150 min/week of combined endurance and resistance exercise plus diet counseling		+	In all subjects
Reinehr 2015 [10]	*n* = 60 Germany 10-15 y-o NW (*n* = 20 (10 boys)), OB (*n* = 40 (20 boys)).	Exercise sessions once per week plus nutrition education for 4–6 weeks		0	In OB children who lost weight
				+	In OB children who did not lose weight
Singhal 2014 [12]	*n* = 85 women (81 Caucasian and Asian, 11 mixed-race, 5 Black) 14–21 y-o. AA (*n* = 38), EA (*n* = 24), NA (*n* = 23).	No intervention		−	In AA
				0	In EA and NA

AA (amenorrheic athletes), CO (Control), CS (Competitive sports), DBP (Dystolic Blood Pressure), EA (eumenorrheic athletes), HRmax (maximum heart rate), HRR (heart rate reserve), IN (Intervention), Ir (irisin), NA (non-athletes), NW (normal weight), OB (obese), OGTT (oral glucose tolerance test), OW (overweight), RM (repetition maximum), UW (underweight) WC (waist circumference), + (positive correlation), − (negative correlation), 0 (no correlation).

**Table 3:** Association of circulating irisin with cardiovascular risk factors and metabolic alterations.

Author	Sample	Insulin	HOMA	Glucose	TG	HDL-c	LDL-c	TC	MS	Leptin	Others
Löffler 2015 [11]	*n* = 105 (46 boys) Germany NW (*n* = 20), OW/OB (*n* = 64) 8–21 y-o and OB (*n* = 58 (23 boys)) 7–17 y-o.	0	0	0	0	0	0	0	N/A	N/A	N/A
Al-Daghri 2014 [28]	*n* = 133 (76 boys) Saudi Arabia 9–15 years y-o. OB (*n* = 30).	0	0	−	0	0	0	0	N/A	0	(+) with ANG II
Reinehr 2015 [10]	*n* = 60 Germany 10–15 y-o NW (*n* = 20 (10 boys)), OB (*n* = 40 (20 boys))	+	+	0	+	−	+	N/A	N/A	N/A	(+) with 2-h OGTT and DBP
Binay 2017 [7]	*n* = 120 Turkey 10–18 y-o NW (*n* =30), OB (*n* = 90).	+	+	+	N/A	N/A	N/A	N/A	N/A	N/A	(+) with SBP in OB
Catli 2016 [5]	*n* = 66 Turkey 8–15 y-o. OB (*n* = 20 (20 boys)), NW (*n* = 30 (16 boys)).	+	+	0	0	−	0	0	N/A	0	N/A
Nigro 2017 [20]	*n* = 27 (19 boys) OB Italy 4–13 y-o, NW (*n* = 13 (4 boys)).	N/A	N/A	N/A	N/A	N/A	N/A	N/A	N/A	N/A	(−) with adiponectin
Blüher 2014 [27]	*n* = 65 OB (35 boys) 7–18 y-o.	0	0	0	N/A	N/A	N/A	N/A	N/A	N/A	No relation with adiponectin, leptin, or resistin
De Meneck 2018 [29]	*n* = 87 (47 boys) Brazi l6–12 y-o. NW (*n* = 63), OW/OB (*n* = 24)	+	+	+	+	−	N/A	0	N/A	N/A	(+) with SBP and DBP in the entire cohort, (+) with EPCs
Viitasalo 2015 [30]	*n* = 444 (247 boys) Finland 6–9 y-o. NW (*n* = 388), OW/OB (*n* = 55).	N/A	N/A	N/A	N/A	N/A	N/A	N/A	N/A	N/A	(+) with unfavorable fatty acid profile
Shim 2018 [9]	*n* = 96 (56 boys) Korea 6 to 10 y-o. NW (*n* = 54), OW (*n* = 16), OB (*n* = 26).	N/A	N/A	−	(−) in OW/OB	0	0	0	−	N/A	(+) with SBP and DBP in OB/OW
Palacios-González 2015 [6]	*n* = 85 (40 boys) Mexico 8–10 y-o. NW (*n* = 25), OW (*n* = 23), OB (*n* = 37).	N/A	N/A	N/A	N/A	N/A	N/A	N/A	N/A	+	N/A

DBP (diastolic blood pressure), HDL-c (high-density lipoprotein cholesterol), LDL-c (low-density lipoprotein cholesterol), N/A (not available), NW (normal weight), OB (obese), OGGT (oral glucose tolerance test), OW (overweight), PBF (percent body fat), SBP (systolic blood pressure), TC (total cholesterol, + (positive correlation), − (negative correlation), 0 (no correlation).

## Abbreviations

UCP-1	uncoupling protein 1
FNDC5	fibronectin type III domain-containing protein 5
VAT	visceral adipose tissue
SAT	subcutaneous adipose tissue
MS	metabolic syndrome
BMI	body mass index
WC	waist circumference
LDL-c	lipoprotein-c
FFM	fat free mass
AAs	amenorrheic athletes
EAs	eumenorrheic athletes
BMD	bone mineral density
NAs	non-athletes
T1DM	type 1 diabetes mellitus
SBP	systolic blood pressure
DBP	diastolic blood pressure
SGA	small for gestational age
AGA	appropriate for gestational age
LGA	large for gestational age
IUGR	intrauterine growth restriction
rhGH	recombinant human growth hormone

## References

[1] Boström P., Wu J., Jedrychowski M.P., Korde A., Ye L., Lo J.C., Rasbach K.A., Boström E.A., Choi J.H., Long J.Z. (2012). A PGC1-α-dependent myokine that drives brown-fat-like development of white fat and thermogenesis. Nature.

[2] Roca-Rivada A., Castelao C., Senin L.L., Landrove M.O., Baltar J., Belen Crujeiras A., Seoane L.M., Casanueva F.F., Pardo M. (2013). FNDC5/irisin is not only a myokine but also an adipokine. PLoS ONE.

[3] Moreno-Navarrete J.M., Ortega F., Serrano M., Guerra E., Pardo G., Tinahones F., Ricart W., Fernandez-Real J.M. (2013). Irisin is expressed and produced by human muscle and adipose tissue in association with obesity and insulin resistance. J. Clin. Endocrinol. Metab..

[4] Elizondo-Montemayor L., Silva-Platas C., Torres-Quintanilla A., Rodríguez-López C., Ruiz-Esparza G.U., Reyes-Mendoza E., Garcia-Rivas G. (2017). Association of Irisin Plasma Levels with Anthropometric Parameters in Children with Underweight, Normal Weight, Overweight, and Obesity. Biomed. Res. Int..

[5] Çatlı G., Küme T., Tuhan H., Anık A., Çalan Ö., Böber E., Abacı A. (2016). Relation of serum irisin level with metabolic and antropometric parameters in obese children. J. Diabetes Complicat..

[6] Palacios-González B., Vadillo-Ortega F., Polo-Oteyza E., Sánchez T., Ancira-Moreno M., Romero-Hidalgo S., Meráz N., Antuna-Puente B. (2015). Irisin levels before and after physical activity among school-age children with different BMI: A direct relation with leptin. Obesity.

[7] Binay Ç., Paketçi C., Güzel S., Samancı N. (2017). Serum Irisin and Oxytocin Levels as Predictors of Metabolic Parameters in Obese Children. J. Clin. Res. Pediatr. Endocrinol..

[8] Jang H.B., Kim H.J., Kang J.H., Park S.I., Park K.H., Lee H.J. (2017). Association of circulating irisin levels with metabolic and metabolite profiles of Korean adolescents. Metabolism.

[9] Shim Y.S., Kang M.J., Yang S., Hwang I.T. (2018). Irisin is a biomarker for metabolic syndrome in prepubertal children. Endocr. J..

[10] Reinehr T., Elfers C., Lass N., Roth C.L. (2015). Irisin and its relation to insulin resistance and puberty in obese children: A longitudinal analysis. J. Clin. Endocrinol. Metab..

[11] Löffler D., Müller U., Scheuermann K., Friebe D., Gesing J., Bielitz J., Erbs S., Landgraf K., Wagner I.V., Kiess W. (2015). Serum irisin levels are regulated by acute strenuous exercise. J. Clin. Endocrinol. Metab..

[12] Singhal V., Lawson E.A., Ackerman K.E., Fazeli P.K., Clarke H., Lee H., Eddy K., Marengi D.A., Derrico N.P., Bouxsein M.L. (2014). Irisin levels are lower in young amenorrheic athletes compared with eumenorrheic athletes and non-athletes and are associated with bone density and strength estimates. PLoS ONE.

[13] Soininen S., Sidoroff V., Lindi V., Mahonen A., Kröger L., Kröger H., Jääskeläinen J., Atalay M., Laaksonen D.E., Laitinen T. (2018). Body fat mass, lean body mass and associated biomarkers as determinants of bone mineral density in children 6–8 years of age—The Physical Activity and Nutrition in Children (PANIC) study. Bone.

[14] Faienza M.F., Brunetti G., Sanesi L., Colaianni G., Celi M., Piacente L., D’Amato G., Schipani E., Colucci S., Grano M. (2018). High irisin levels are associated with better glycemic control and bone health in children with Type 1 diabetes. Diabetes Res. Clin. Pract..

[15] Perakakis N., Triantafyllou G.A., Fernandez-Real J.M., Huh J.Y., Park K.H., Seufert J., Mantzoros C.S. (2017). Physiology and role of irisin in glucose homeostasis. Nat. Rev. Endocrinol..

[16] Huh J.Y., Panagiotou G., Mougios V., Brinkoetter M., Vamvini M.T., Schneider B.E., Mantzoros C.S. (2012). FNDC5 and irisin in humans: I. Predictors of circulating concentrations in serum and plasma and II. mRNA expression and circulating concentrations in response to weight loss and exercise. Metabolism.

[17] Pardo M., Crujeiras A.B., Amil M., Aguera Z., Jimenez-Murcia S., Banos R., Botella C., de la Torre R., Estivill X., Fagundo A.B. (2014). Association of irisin with fat mass, resting energy expenditure, and daily activity in conditions of extreme body mass index. Int. J. Endocrinol..

[18] Crujeiras A.B., Zulet M.A., Lopez-Legarrea P., de la Iglesia R., Pardo M., Carreira M.C., Martínez J.A., Casanueva F.F. (2014). Association between circulating irisin levels and the promotion of insulin resistance during the weight maintenance period after a dietary weight-lowering program in obese patients. Metabolism.

[19] Stengel A., Hofmann T., Goebel-Stengel M., Elbelt U., Kobelt P., Klapp B.F. (2013). Circulating levels of irisin in patients with anorexia nervosa and different stages of obesity-correlation with body mass index. Peptides.

[20] Nigro E., Scudiero O., Ludovica Monaco M., Polito R., Schettino P., Grandone A., Perrone L., Miraglia Del Giudice E., Daniele A. (2017). Adiponectin profile and Irisin expression in Italian obese children: Association with insulin-resistance. Cytokine.

[21] Anastasilakis A.D., Polyzos S.A., Makras P., Gkiomisi A., Bisbinas I., Katsarou A., Filippaios A., Mantzoros C.S. (2014). Circulating irisin is associated with osteoporotic fractures in postmenopausal women with low bone mass but is not affected by either teriparatide or denosumab treatment for 3 months. Osteoporos. Int..

[22] Yan J., Liu H.J., Guo W.C., Yang J. (2018). Low serum concentrations of Irisin are associated with increased risk of hip fracture in Chinese older women. Jt. Bone Spine.

[23] Palermo A., Strollo R., Maddaloni E., Tuccinardi D., D’Onofrio L., Briganti S.I., Defeudis G., De Pascalis M., Lazzaro M.C., Colleluori G. (2015). Irisin is associated with osteoporotic fractures independently of bone mineral density, body composition or daily physical activity. Clin. Endocrinol..

[24] Lee P., Brychta R.J., Collins M.T., Linderman J., Smith S., Herscovitch P., Millo C., Chen K.Y., Celi F.S. (2013). Cold-activated brown adipose tissue is an independent predictor of higher bone mineral density in women. Osteoporos. Int..

[25] Ponrartana S., Aggabao P.C., Hu H.H., Aldrovandi G.M., Wren T.A., Gilsanz V. (2012). Brown adipose tissue and its relationship to bone structure in pediatric patients. J. Clin. Endocrinol. Metab..

[26] Blizzard LeBlanc D.R., Rioux B.V., Pelech C., Moffatt T.L., Kimber D.E., Duhamel T.A., Dolinsky V.W., McGavock J.M., Senechal M. (2017). Exercise-induced irisin release as a determinant of the metabolic response to exercise training in obese youth: The EXIT trial. Physiol. Rep..

[27] Bluher S., Panagiotou G., Petroff D., Markert J., Wagner A., Klemm T., Filippaios A., Keller A., Mantzoros C.S. (2014). Effects of a 1-year exercise and lifestyle intervention on irisin, adipokines, and inflammatory markers in obese children. Obesity.

[28] Al-Daghri N.M., Alkharfy K.M., Rahman S., Amer O.E., Vinodson B., Sabico S., Piya M.K., Harte A.L., McTernan P.G., Alokail M.S. (2014). Irisin as a predictor of glucose metabolism in children: Sexually dimorphic effects. Eur. J. Clin. Investig..

[29] De Meneck F., Victorino de Souza L., Oliveira V., do Franco M.C. (2018). High irisin levels in overweight/obese children and its positive correlation with metabolic profile, blood pressure, and endothelial progenitor cells. Nutr. Metab. Cardiovasc. Dis..

[30] Viitasalo A., Atalay M., Pihlajamaki J., Jaaskelainen J., Korkmaz A., Kaminska D., Lindi V., Lakka T.A. (2015). The 148 M allele of the PNPLA3 is associated with plasma irisin levels in a population sample of Caucasian children: The PANIC Study. Metabolism.

[31] Viitasalo A., Agren J., Venalainen T., Pihlajamaki J., Jaaskelainen J., Korkmaz A., Atalay M., Lakka T.A. (2016). Association of plasma fatty acid composition with plasma irisin levels in normal weight and overweight/obese children. Pediatr. Obes..

[32] Steffen L.M., Vessby B., Jacobs D.R., Steinberger J., Moran A., Hong C.P., Sinaiko A.R. (2008). Serum phospholipid and cholesteryl ester fatty acids and estimated desaturase activities are related to overweight and cardiovascular risk factors in adolescents. Int. J. Obes..

[33] Lawson E.A., Ackerman K.E., Slattery M., Marengi D.A., Clarke H., Misra M. (2014). Oxytocin secretion is related to measures of energy homeostasis in young amenorrheic athletes. J. Clin. Endocrinol. Metab..

[34] Al-Daghri N.M., Al-Attas O.S., Alokail M.S., Alkharfy K.M., Yousef M., Vinodson B., Amer O.E., Alnaami A.M., Sabico S., Tripathi G. (2014). Maternal inheritance of circulating irisin in humans. Clin. Sci..

[35] Hernandez-Trejo M., Garcia-Rivas G., Torres-Quintanilla A., Laresgoiti-Servitje E. (2016). Relationship between Irisin Concentration and Serum Cytokines in Mother and Newborn. PLoS ONE.

[36] Karras S.N., Polyzos S.A., Newton D.A., Wagner C.L., Hollis B.W., Ouweland J.V.D., Dursun E., Gezen-Ak D., Kotsa K., Annweiler C. (2018). Adiponectin and vitamin D-binding protein are independently associated at birth in both mothers and neonates. Endocrine.

[37] Joung K.E., Park K.H., Filippaios A., Dincer F., Christou H., Mantzoros C.S. (2015). Cord blood irisin levels are positively correlated with birth weight in newborn infants. Metabolism.

[38] Keles E., Turan F.F. (2016). Evaluation of cord blood irisin levels in term newborns with small gestational age and appropriate gestational age. SpringerPlus.

[39] Pavlova T., Zlamal F., Tomandl J., Hodicka Z., Gulati S., Bienertova-Vasku J. (2018). Irisin Maternal Plasma and Cord Blood Levels in Mothers with Spontaneous Preterm and Term Delivery. Dis. Mark..

[40] Baka S., Malamitsi-Puchner A., Boutsikou T., Boutsikou M., Marmarinos A., Hassiakos D., Gourgiotis D., Briana D.D. (2015). Cord blood irisin at the extremes of fetal growth. Metabolism.

[41] Padoan A., Rigano S., Ferrazzi E., Beaty B.L., Battaglia F.C., Galan H.L. (2004). Differences in fat and lean mass proportions in normal and growth-restricted fetuses. Am. J. Obstet. Gynecol..

[42] Van de Lagemaat M., Rotteveel J., Lafeber H.N., van Weissenbruch M.M. (2014). Lean mass and fat mass accretion between term age and 6 months post-term in growth-restricted preterm infants. Eur. J. Clin. Nutr..

[43] Verkauskiene R., Beltrand J., Claris O., Chevenne D., Deghmoun S., Dorgeret S., Alison M., Gaucherand P., Sibony O., Levy-Marchal C. (2007). Impact of fetal growth restriction on body composition and hormonal status at birth in infants of small and appropriate weight for gestational age. Eur. J. Endocrinol..

[44] Ornoy A. (2011). Prenatal origin of obesity and their complications: Gestational diabetes, maternal overweight and the paradoxical effects of fetal growth restriction and macrosomia. Reprod. Toxicol..

[45] Bakal U., Aydin S., Sarac M., Kuloglu T., Kalayci M., Artas G., Yardim M., Kazez A. (2016). Serum, Saliva, and Urine Irisin with and Without Acute Appendicitis and Abdominal Pain. Biochem. Insights.

[46] Elhady M., Youness E.R., Gafar H.S., Abdel Aziz A., Mostafa R.S.I. (2018). Circulating irisin and chemerin levels as predictors of seizure control in children with idiopathic epilepsy. Neurol. Sci..

[47] Zhao Y.T., Wang H., Zhang S., Du J., Zhuang S., Zhao T.C. (2016). Irisin Ameliorates Hypoxia/Reoxygenation-Induced Injury through Modulation of Histone Deacetylase 4. PLoS ONE.

[48] Mazur-Bialy A.I., Pochec E., Zarawski M. (2017). Anti-Inflammatory Properties of Irisin, Mediator of Physical Activity, Are Connected with TLR4/MyD88 Signaling Pathway Activation. Int. J. Mol. Sci..

[49] Wikiera B., Zawadzka K., Laczmanski L., Sloka N., Bolanowski M., Basiak A., Noczynska A., Daroszewski J. (2017). Growth Hormone Treatment Increases Plasma Irisin Concentration in Patients with Turner Syndrome. Horm. Metab. Res..

